# Hypermethylation of the *hMLH1* gene promoter in solitary and multiple gastric cancers with microsatellite instability

**DOI:** 10.1038/sj.bjc.6600076

**Published:** 2002-02-12

**Authors:** K Sakata, G Tamura, Y Endoh, K Ohmura, S Ogata, T Motoyama

**Affiliations:** Department of Pathology, Yamagata University School of Medicine, Iida-nishi 2-2-2, Yamagata 990-9585, Japan

**Keywords:** gastric cancer, hMLH1, hypermethylation, multiple cancer

## Abstract

Human cancers with a high frequency microsatellite instability phenotype develop due to defects in DNA mismatch repair genes. Silencing of a DNA mismatch repair gene, *hMLH1* gene, by promoter hypermethylation is a frequent cause of the microsatellite instability-H phenotype. Using methylation specific PCR we investigated the methylation status of the *hMLH1* gene promoter in 17 solitary gastric cancers (12 microsatellite instability-H and five microsatellite stable tumours from 17 patients), and 13 multiple gastric cancers (eight microsatellite instability-H, one low frequency microsatellite instability-L and four microsatellite stable tumours from five patients) and also examined non-cancerous gastric mucosa both adjacent to and distant from each tumour. Expression of hMLH1 protein was evaluated by immunohistochemistry. All microsatellite instability-H tumours (20 out of 20) had evidence of methylation of *hMLH1* promoter, whereas only one out of 10 microsatellite instability-L and microsatellite stable tumours did (*P*<0.0000005), and the methylation status correlated with hMLH1 protein expression (*P*<0.000003). Furthermore, methylation of the *hMLH1* promoter was detected in 50% (6 out of 12) and 63% (5 out of 8) of non-cancerous gastric mucosa samples adjacent to, and in 33% (4 out of 12) and 40% (2 out of 5) of those obtained from distant portion of, solitary and multiple cancers with microsatellite instability-H. Thus both solitary and multiple gastric cancers with microsatellite instability-H have evidence of similar high levels of *hMLH1* promoter hypermethylation in the surrounding non-cancerous tissue. Hypermethylation of the *hMLH1* promoter occurs in non-cancerous gastric mucosa of microsatellite instability-H tumours and may increase the risk of subsequent neoplasia.

*British Journal of Cancer* (2002) **86**, 564–567. DOI: 10.1038/sj/bjc/6600076 www.bjcancer.com

© 2002 Cancer Research UK

## 

Microsatellite instability (MSI) due to defects in mismatch repair genes such as *hMLH1* and *hMSH2* is now widely recognized as an important mechanism in tumorigenesis (Aaltonen *et al*, 1993; Ionov *et al*, 1993; Thibodeau *et al*, 1996; Fleisher *et al*, 1999; Kang *et al*, 1999; Leung *et al*, 1999; Suzuki *et al*, 1999; Toyota *et al*, 1999a). MSI is reportedly present in 15–33% of solitary gastric cancers, although mutations of the *hMLH1* or *hMSH2* genes are rare in sporadic gastric cancers (Chong *et al*, 1994; Mironov *et al*, 1994; Strickler *et al*, 1994; Tamura *et al*, 1996). Hypermethylation of promoter region CpG islands is a common mechanism by which tumour suppressor or tumour-related genes, and DNA mismatch repair genes are inactivated (Herman *et al*, 1995, 1998; Kane *et al*, 1997; Baylin *et al*, 1998; Jones and Laird, 1999). Aberrant DNA methylation of promoter region CpG islands of several genes, including *retinoblastoma* (*Rb*), *von Hippel-Lindau* (*VHL*), *p16*, *p15*, *APC*, *E-cadherin* and *hMLH1* has been reported in human cancers, and silencing of *hMLH1* by promoter hypermethylation is the major causative event in the development of human cancers with MSI phenotype, including gastric cancers (Graff *et al*, 1997; Kane *et al*, 1997; Herman *et al*, 1998; Fleisher *et al*, 1999; Kang *et al*, 1999; Leung *et al*, 1999; Suzuki *et al*, 1999; Tamura *et al*, 2000; Tsuchiya *et al*, 2000).

To clarify the role of hypermethylation of the *hMLH1* gene promoter in the development of multiple gastric cancers, we compared both the methylation status and expression of the *hMLH1* gene, in solitary and multiple gastric cancers with and without MSI. In addition, we examined the methylation status of the *hMLH1* gene in non-cancerous gastric mucosa adjacent to and distant from each tumour.

## MATERIALS AND METHODS

### Samples and DNA extraction

Thirty gastric adenocarcinomas, including 17 solitary tumours from 17 patients and 13 multiple tumours from five patients were studied. The patients ranged in age from 59 to 84 years (average 72 years). We also examined non-cancerous gastric tissue both adjacent to (at a distance of 2 mm) and distant from (at a distance of 5 cm or surgical margin) each tumour. The tumours were histologically differentiated adenocarcinomas comprising 28 early cancers (depth of invasion limited to the mucosa or submucosa) and two advanced cancers. Specimens were fixed in 10% buffered formalin. The carcinomas were cut serially into 5 mm slices in parallel with the lesser curvature and then embedded in paraffin. From one block that included the maximum diameter of the tumour, we prepared two sets of 3 and 10 μm thick serial sections. The 3 μm thick sections were used for haematoxylin-eosin and immunohistochemical stainings, and each set of the 10 μm thick sections for DNA extraction of cancerous or non-cancerous DNA. To avoid contamination, either of cancerous or non-cancerous the area was carefully dissected using disposable scalpels by a reference to the serial haematoxylin-eosin stained sections under the microscope. DNA extraction was as described by Goelz *et al* (1985). All samples had been previously screened for MSI using 12 microsatellite markers, i.e. D2S115, D4S404, D5S178, IL9, D6S265, D7S490, D11S900, MYH6, TP53, D17S1176, D18S46, and D21S1407, and defined as MSI-H if there were more than 30% unstable loci, MSI-L less than 30% unstable loci, and microsatellite stable (MSS) no unstable loci (Ohmura *et al*, 2000; Ogata *et al*, 2001). Our samples constituted of 20 (12 solitary and eight multiple) MSI-H tumours, 1 MSI-L tumour and nine (five solitary and four multiple) MSS tumours.

### Methylation-specific PCR

*hMLH1* gene promoter methylation patterns were determined by methylation specific PCR (MSP), as described previously (Fleisher *et al*, 1999). MSP distinguishes unmethylated from methylated alleles of a given gene based on sequence changes that are produced following bisulfite treatment of DNA, which converts unmethylated cytosines to uracils, while leaving methylated cytosines unaffected. Subsequent PCR using primers specific to sequences that correspond to either methylated or unmethylated *hMLH1* gene promoter DNA was performed. The primer sequences of *hMLH1* for the unmethylated reaction were 5′-TTT TGA TGT AGA TGT TTT ATT AGG GTT GT-3′ (sense) and 5′-ACC ACC TCA TCA TAA CTA CCC ACA-3′ (antisense), whereas for the methylated reaction they were 5′-ACG TAG ACG TTT TAT TAG GGT CGC-3′ (sense) and 5′-CCT CAT CGT AAC TAC CCG CG-3′ (antisense) (Fleisher *et al*, 1999). Briefly, 2 μg of genomic DNA was denatured by treatment with NaOH and modified by sodium bisulfite. DNA samples were then purified using a Wizard DNA purification resin (Promega, Madison, WI, USA), treated with NaOH, precipitated with ethanol, and resuspended in 30 μl water. Modified DNA was amplified in a total volume of 20 μl using GeneAmp PCR Gold Buffer (PE Applied Biosystems, Foster City, CA, USA) containing 1.0 mM MgCl_2_, 20 μM of each primer, 0.2 mM dNTPs, and 1 unit of Taq polymerase (AmpliTaq Gold DNA Polymerase, PE Applied Biosystems). After activation of the Taq polymerase at 95°C for 10 min, PCR was performed in a thermal cycler (GeneAmp 9700, PE Applied Biosystems) for 35 cycles, each cycle consisting of denaturation at 95°C for 15 s, annealing at 55°C for 15 s, and extension at 72°C for 30 s, followed by a final 7 min extension at 72°C. The PCR products were then loaded onto a non-denaturing 6% polyacrylamide gel, stained with ethidium bromide, and visualized under UV illumination.

### Immunohistochemistry

Immunohistochemistry was performed on formalin-fixed, paraffin embedded sections using a standard labelled streptavidin-biotin system (Nichirei, Tokyo, Japan). Mouse monoclonal antibody to the *hMLH1* gene product, G168-728 (PharMingen, San Diego, CA, USA), was used at 1 : 50 dilution after antigen retrieval by microwave.

### Statistical analysis

Statistical comparisons were performed using Fisher's exact test. *P* values <0.05 were considered significant.

## RESULTS

**Figure 1:**
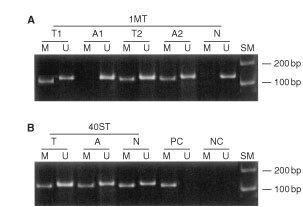
Representative results from methylation-specific PCR (MSP) of the *hMLH1* gene promoter in multiple (**A**) and solitary (**B**) gastric cancers. The presence of PCR product in lanes marked M indicates hypermethylated *hMLH1* product, lanes marked U indicate unmethylated *hMLH1*. T, Tumour DNA; A, normal mucosa adjacent to tumour; N, normal mucosa from the surgical margin; PC, positive control; NC, negative control; SM, size marker.

Hypermethylation of the *hMLH1* gene promoter was detected in all solitary (12 out of 12) and multiple (8 out of 8) gastric cancers with MSI-H ( Figure 1

and Table 1

**Table 1:**
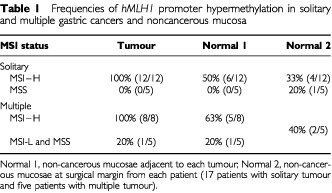
Frequencies of *hMLH1* promoter hypermethylation in solitary and multiple gastric cancers and noncancerous mucosa

). Methylated alleles were found in none (0 out of 5) of solitary and 20% (1 out of 5) of multiple MSI-L and MSS tumours (Table 1). The MSI-L tumour did not have methylated *hMLH1* gene promoter alleles. The methylation status of the *hMLH1* promoter was significantly correlated with MSI status (*P*<0.0000005). Methylated *hMLH1* alleles were also present in 50% (6 out of 12) and 63% (5 out of 8) of samples of non-cancerous gastric mucosa adjacent to, and in 33% (4 out of 12) and 40% (2 out of 5) samples of non-cancerous gastric mucosa distant from solitary and multiple gastric cancers with MSI-H (Table 1). In contrast, methylated alleles were infrequently present in none (0 out of 5) of non-cancerous mucosa adjacent to, and in 20% (1 out of 5) of non-cancerous mucosa distant from solitary gastric cancers with MSS (Table 1). Most of the methylated tumours (12 out of 12 solitary tumours and 7 out of 9 (78%) of multiple tumours) showed an apparent loss of hMLH1 protein expression ( Figure 2

**Figure 2:**
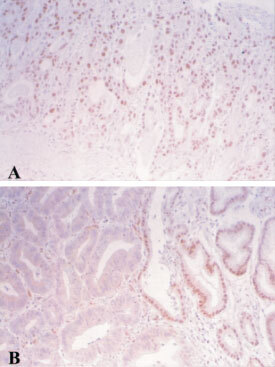
Immunohistochemical staining for hMLH1 protein expression in gastric cancers with unmethylated (**A**) or hypermethylated (**B**) gene promoters. (**A**) Nuclear staining of hMLH1 in a MSS tumour without promoter hypermethylation (intramucosal well differentiated tubular adenocarcinoma). (**B**) Loss of hMLH1 expression in a MSI-H tumour with promoter hypermethylation (left, intramucosal well differentiated tubular adenocarcinoma; right, intestinal metaplastic mucosa exhibiting hMLH1 expression).

). All nine unmethylated tumours (five solitary and four multiple tumours) and their surrounding non-cancerous mucosa had a normal expression level of hMLH1 protein. The methylation status of the *hMLH1* promoter was significantly correlated with protein expression in solitary and multiple tumours (*P*<0.000003, Table 2

**Table 2:**
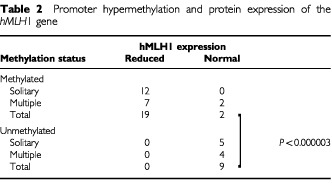
Promoter hypermethylation and protein expression of the *hMLH*1 gene

).

## DISCUSSION

MSI is found in 15–33% of sporadic gastric cancers, a higher incidence than that seen in other types of sporadic human cancers (Chong *et al*, 1994; Mironov *et al*, 1994; Strickler *et al*, 1994; Tamura *et al*, 1996; Ohmura *et al*, 2000). Furthermore, multiple gastric cancers have a higher incidence of MSI than solitary gastric cancers, although few studies have focused on the relationship between multiple gastric cancers and MSI (Nakashima *et al*, 1995; Yamashita *et al*, 2000; Ogata *et al*, 2001). MSI has been observed in 58.3% (7 out of 12) – 80% (4 out of 5) of multiple gastric cancer patients, and in 30.3% (10 out of 33) – 71.4% (10 out of 14) of individual tumours (Nakashima *et al*, 1995; Yamashita *et al*, 2000; Ogata *et al*, 2001). These data suggest that MSI may play a more important role in the development of multiple rather than solitary gastric cancers. Recent studies suggest that silencing of the *hMLH1* gene by promoter hypermethylation is a major causative event in the development of human gastric cancers with MSI (Fleisher *et al*, 1999, 2001; Kang *et al*, 1999; Leung *et al*, 1999; Suzuki *et al*, 1999). *hMLH1* promoter hypermethylation was observed in 62.5–100% of sporadic gastric cancers with MSI-H (Fleisher *et al*, 1999, 2001; Kang *et al*, 1999; Leung *et al*, 1999; Suzuki *et al*, 1999). The majority of these tumours also exhibited loss of hMLH1 protein expression. In our present study, *hMLH1* promoter hypermethylation occurred in both solitary (12 out of 12) and multiple gastric cancers (8 out of 8) exhibiting MSI-H, and all but two tumour samples also had an apparent loss of hMLH1 protein expression. Toyota *et al* (1999b) examined the methylation status of multiple CpG islands in the DNA from normal gastric mucosa adjacent to gastric tumours with a CpG island methylator phenotype, and found that hypermethylation was rarely detected. Similar findings of *hMLH1* gene promoter were reported by other investigators (Suzuki *et al*, 1999; Leung *et al*, 2001). In our present study, however, methylation of the *hMLH1* promoter was detected in non-cancerous mucosa adjacent to both solitary (6 out of 12, 50%) and multiple (5 out of 8, 63%) gastric tumours with MSI-H. Similarly to our present results, Guo *et al* (2001) has recently reported a high frequency (40%; 4 out of 10) of *hMLH1* methylation in non-cancerous mucosa adjacent to gastric cancer showing MSI-H. The exact cause of these discrepancies among reports remains uncertain. However, because hypermethylation originates within the flanking regions of the CpG islands (Graff *et al*, 1997) and the more 3′ region of *hMLH1* promoter than we studied displayed a higher degree of correlation with MSI status (Deng *et al*, 1999; Nakagawa *et al*, 2001), it is possible that *hMLH1* promoter was not yet fully methylated (or silenced) in non-cancerous mucosa in which reduction of hMLH1 expression was inconspicuous. Alternatively, MSI-H gastric cancers may develop through clonal expansion of fully methylated cells showing loss of hMLH1. Such a finding has been previously reported in colorectal tissue (Kuismanen *et al*, 1999).

Multiple gastric cancers are frequently found in the elderly, and the incidence of gastric cancers with MSI-H also correlates with age (Esaki *et al*, 1987; Nakashima *et al*, 1995; dos Santos *et al*, 1996; Halling *et al*, 1999). Furthermore, hypermethylation of the promoters of several tumour-related genes is also increased with age (Ahuja *et al*, 1997, 1998; Kane *et al*, 1997; Veigl *et al*, 1998). Thus, age-related methylation has the potential to behave as a mutator process resulting in the simultaneous silencing of multiple tumour related genes in aging tissues (Lipkin, 1988; Issa, 2000). While the mechanism of age-related methylation is not known, it is clear that age-related methylation only affects a subset of genes, suggesting a gene-specific susceptibility to this process (Issa, 2000). Several factors have been suggested to modulate this process, such as exogenous carcinogens, endogenously generated reactive oxygen species, and genetic differences in individuals' susceptibility to age-related methylation (Issa, 2000). Although we found frequent hypermethylation of the *hMLH1* gene promoter in non-cancerous mucosa adjacent to, or distant from solitary and multiple gastric cancers with MSI-H, methylated alleles were rarely detected in similar tissues from patients with solitary gastric cancer of the MSS phenotype. We conclude that hypermethylation of the *hMLH1* gene promoter occurs in the non-cancerous mucosa which varies significantly among individuals and may lead to the development of MSI-H gastric cancer. Such methylation in non-cancerous mucosa may also increase the risk of subsequent neoplasia.
